# Electrical impedance tomography assessment of bronchoalveolar lavage-associated lung derecruitment and its related factors: a single-center retrospective study

**DOI:** 10.3389/fphys.2026.1900825

**Published:** 2026-09-04

**Authors:** Aijie Yang, Zhanqi Zhao, Hui Yuan, Yuan Shen, Li Wang, Yuhua Wu, Bin Guan, Kailu Zhang, Meng Dai, Wei Gao

**Affiliations:** 1 Department of Anesthesiology, The First Affiliated Hospital of Xi’an Jiaotong University, Xi’an, China; 2 Department of Anesthesiology, Xi’an Hospital of Traditional Chinese Medicine, Xi’an, China; 3 School of Biomedical Engineering, Guangzhou Medical University, Guangzhou, China; 4 School of Public Health, Health Science Centre of Xi’an Jiaotong University, Xi’an, China; 5 Department of Respiratory Medicine, The First Affiliated Hospital of Xi’an Jiaotong University, Xi’an, China; 6 Department of Imaging, Xi’an Hospital of Traditional Chinese Medicine, Xi’an, China; 7 Department of Biomedical Engineering, The Fourth Military Medical University, Xi’an, China

**Keywords:** atelectasis, bronchoalveolar lavage, electrical impedance tomography, lung derecruitment, regional ventilation

## Abstract

**Background:**

Bronchoalveolar lavage (BAL) may cause regional lung derecruitment during bronchoscopy. However, this effect may be underestimated by conventional oxygenation monitoring, especially under general anesthesia and mechanical ventilation. This study aimed to assess BAL-associated lung derecruitment using electrical impedance tomography (EIT) and to explore related clinical, radiological, and physiological factors.

**Methods:**

This single-center retrospective observational study included adult patients who underwent BAL with periprocedural EIT monitoring between March 2024 and October 2025. EIT data were analyzed at three time points: before BAL, immediately after BAL, and after completion of the procedure. The primary outcome was residual end-expiratory lung impedance loss after BAL. Secondary outcomes included changes in defect score, global inhomogeneity index, center of ventilation, regional ventilation delay, respiratory mechanics, and oxygen saturation. Linear regression was used to identify factors associated with residual EELI loss.

**Results:**

Among 405 screened patients, 286 met the inclusion criteria, and 277 were finally analyzed. BAL was associated with a significant decrease in EELI and increases in defect score and global inhomogeneity index, indicating lung derecruitment and more heterogeneous ventilation distribution. Driving pressure increased and dynamic compliance decreased after BAL, while SpO_2_ showed no significant change. In multivariable analysis, preoperative atelectasis was independently associated with greater residual EELI loss, with an adjusted β coefficient of 1.71 (95% CI 0.41–3.00, P = 0.010). Baseline defect score was also independently associated with residual EELI loss, with an adjusted β coefficient of 0.51 (95% CI 0.07–0.95, P = 0.025). In contrast, total BAL instilled volume, number of BAL cycles, and bronchoscope insertion duration were not significantly associated with residual EELI loss.

**Conclusions:**

BAL was associated with immediate lung derecruitment and worsening of regional ventilation distribution under general anesthesia and mechanical ventilation, despite stable SpO_2_. Preoperative atelectasis and baseline regional ventilation defects were independently related to incomplete lung volume recovery after BAL. EIT may help identify patients at higher risk of BAL-associated lung derecruitment and support individualized respiratory management during bronchoscopy.

## 1 Introduction

Bronchoalveolar lavage (BAL) is an important method widely used during diagnostic and therapeutic bronchoscopy (Bruni et al., 2026). It is commonly applied for etiological, cytological, and inflammatory assessment in patients with pulmonary infection, interstitial lung disease, lung cancer, chronic airway disease, atelectasis, and other lung disorders (de Lima et al., 2018). In addition to its diagnostic value, BAL may also have therapeutic effects in selected patients by removing airway secretions, mucus plugs, and inflammatory exudates. However, many factors during BAL reduce alveolar aeration, including fluid instillation, fluid recovery, airway occupation by the bronchoscope, negative suction pressure, transient interruption of ventilation, and changes in airway pressure (Li et al., 2025). Small airway closure and regional lung derecruitment may occur, and be more pronounced in patients with pre-existing pulmonary consolidation, infection, atelectasis, airway stenosis, or diffuse parenchymal lung disease (Hakkarainen et al., 2023).

Conventional periprocedural monitoring mainly relies on pulse oxygen saturation (SpO_2_), tidal volume (VT), airway pressure, driving pressure (ΔP), lung compliance, and arterial oxygenation indices. These variables reflect global oxygenation and respiratory mechanics, but not regional differences in ventilation (Mauri, 2021). Under general anesthesia or deep sedation, patients often receive a high fraction of inspired oxygen (FiO_2_). As a result, SpO_2_ may remain high even when regional ventilation defect is presented after BAL. Early or occult lung derecruitment may therefore be undetected. Thus, conventional monitoring alone may underestimate the immediate effect of BAL on regional ventilation.

Electrical impedance tomography (EIT) is a non-invasive, radiation-free bedside imaging technique that enables real-time assessment of regional lung ventilation. By detecting impedance changes on the thoracic surface, EIT can provide dynamic information on changes in end-expiratory lung volume and ventilation distribution during mechanical ventilation (Bayat et al., 2025). Previous animal and clinical studies have shown that BAL may cause transient regional lung derecruitment, ventilation redistribution, and side-dependent ventilation loss (Frerichs et al., 2019; Franchineau et al., 2021). EIT has also been used to guide individualized bronchoscopic therapy and led to improve in dorsal ventilation, respiratory mechanics, oxygenation, and ventilator weaning (Fu et al., 2020; Xiong et al., 2026). Nevertheless, current evidence remains limited, especially regarding the immediate physiological effects of BAL in mechanically ventilated adult patients. Further investigation is needed to clarify the short-term regional ventilation changes associated with BAL and their clinical implications.

This study aimed to characterize the short-term regional lung function changes during the periprocedural period of BAL and to identify factors related to incomplete lung volume recovery after BAL.

## 2 Materials and methods

### 2.1 Study design and patients

This was a single-center, retrospective observational study based on prospectively recorded clinical and EIT data. Adult patients who underwent BAL with periprocedural EIT monitoring at our center between March 2024 and October 2025 were included. The study aimed to assess dynamic changes in lung volume and regional ventilation distribution before and after BAL, and to identify factors associated with residual lung derecruitment and ventilation deterioration after BAL.

The study was approved by the institutional ethics committee (XJTU1AF2021LSL–012). Because this was a retrospective study and all data were obtained from routine clinical care and periprocedural monitoring, the requirement for informed consent was waived by the ethics committee.

### 2.2 Inclusion and exclusion criteria

Inclusion criteria::

- age ≥18 years;
- underwent diagnostic or therapeutic bronchoscopic BAL;
- completed continuous periprocedural EIT monitoring;
- had complete EIT data at the predefined key time points;
- had available clinical data, respiratory mechanics, and preoperative chest CT data.

Excluded criteria:

- contraindications to EIT monitoring, or inability to place the thoracic electrode belt;
- severe chest wall deformity, open chest injury, pneumothorax, massive pleural effusion, or other conditions affecting EIT signal interpretation;
- severe periprocedural hemodynamic instability preventing completion of the planned BAL procedure or EIT monitoring;
- missing key outcome variables (including EIT data at the main time points);
- incomplete clinical data or poor data quality deemed unsuitable for analysis.

### 2.3 Anesthesia, respiratory support, and BAL procedure

All patients were non-critically ill, scheduled for elective diagnostic evaluation, and none required mechanical ventilation prior to the procedure. Standardized anesthesia and surgical protocols were applied uniformly. Intraoperative monitoring included EIT-derived ventilatory parameters, electrocardiography, heart rate, non-invasive blood pressure, SpO_2_, end-tidal carbon dioxide partial pressure, and respiratory mechanics. Anesthesia was induced with propofol (1–2 mg·kg^-1^), remifentanil (3–4 µg·kg^-1^·min^-1^), and mivacurium chloride (0.1 mg·kg^-1^), preceded by premedication with penehyclidine and ondansetron. After loss of consciousness, a laryngeal mask airway was inserted, and volume-controlled ventilation was initiated using an anesthesia machine (Dräger Fabius GS, Dräger Medical GmbH, Lübeck, Germany), with a tidal volume of 8 mL·kg^-1^, respiratory rate of 20 breaths·min^-1^, fraction of inspired oxygen (FiO_2_) of 60%, and an inspiratory-to-expiratory ratio of 1:2. Anesthesia was maintained with propofol (4–6 mg·kg^-1^·h^-1^) and remifentanil (0.1–0.4 µg·kg^-1^·min^-1^). The laryngeal mask was removed once spontaneous breathing normalized; patients were transferred to the post-anesthesia care unit and subsequently discharged to the ward or directly from the unit after achieving a Steward recovery score of 6.

BAL was performed by specialized respiratory physicians following a unified institutional protocol, with the lavage site determined by preoperative imaging. Each cycle involved instillation of 20 mL of sterile saline (25 °C) via the bronchoscope, followed by suction recovery; this was repeated 3 to 5 times per patient. Since accurate, continuous recording of recovered lavage volume was not feasible routinely, retained volume was excluded from analysis.

Perioperative clinical data—including demographics, smoking history, underlying lung diseases, chest CT findings, BAL indications, anesthetic and ventilatory parameters, vital signs, and procedural variables—were extracted from electronic and paper records. All entries were independently cross-validated by two data coordinators before the final dataset was locked.

### 2.4 EIT measurements

**Figure 1:**
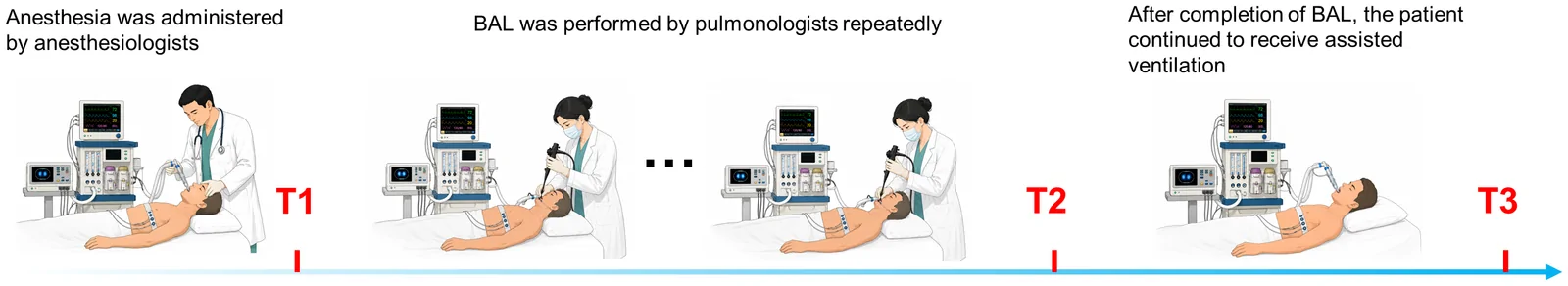
The time points of EIT observation. BAL, bronchoalveolar lavage.

EIT monitoring was routinely performed using an EIT system (VenTOM-100, MidasMED Biomedical Technology, Suzhou, China) with a standard 16-electrode thoracic belt, which acquired impedance images continuously at 20 Hz. which acquired impedance images continuously at 20 Hz. Belt size was selected according to chest circumference, and the belt was placed at the fourth or fifth intercostal space, with positioning adjusted to avoid wounds, dressings, or drainage tubes, and electrode contact quality was verified. Once acceptable signals were obtained, the belt remained in place throughout the monitoring period. Lung impedance data were continuously recorded before, during, and after BAL. All EIT data analyses were performed using MATLAB (2023a, MathWorks, USA), and ventilator parameters were synchronized with EIT signals by matching time stamps referenced to the hospital network time server. As shown in **Figure 1**, EIT measurements were obtained at three predefined procedural time points. T1 was planned approximately 10 min after laryngeal mask insertion and before the initiation of BAL, after the patient’s respiratory and circulatory status had stabilized. T2 was planned approximately 1 min after completion of BAL while the bronchoscope remained in place. T3 was planned approximately 20 min after BAL completion, after bronchoscope removal and while the patient remained under mechanical ventilation. These procedural definitions and target intervals were applied consistently to all patients. Because the ventilation mode, airway management, and oxygen condition were relatively stable after anesthesia, T1 was used as the baseline time point to assess BAL-associated changes in lung volume and regional ventilation distribution.

EIT-derived variables were as follows (Bilir et al., 2026).

End-expiratory lung impedance (EELI) was defined as the impedance value within the predefined lung region at the end of expiration relative to the reference state. EELI is a relative EIT-derived measure that correlates with changes in end-expiratory lung volume but does not provide an absolute measurement of lung volume. Changes in EELI between the predefined procedural time points were therefore used to assess changes in end-expiratory lung aeration. A decrease in EELI indicated a reduction in end-expiratory lung volume, suggesting loss of aeration or lung derecruitment, whereas an increase indicated recovery or gain of end-expiratory lung volume (Hinz et al., 2003; Scaramuzzo et al., 2024).

The ventilation defect score (DS) was calculated based on the four-quadrant ventilation distribution on EIT, as previously described for EIT-based ventilation defect assessment (He et al., 2021; Yang et al., 2023; Frerichs et al., 2024). The lung ventilation image was divided into four quadrants: upper right (UR), upper left (UL), lower right (LR), and lower left (LL). The percentage of ventilation in each quadrant relative to total lung ventilation was calculated. A quadrant was assigned 0 points if its ventilation proportion was ≥15%, 1 point if it was 10%–15%, and 2 points if it was <10% (He et al., 2021). The total DS was the sum of the scores from the four quadrants, ranging from 0 to 6. A score of 0 indicates homogeneous ventilation across all quadrants (each ≥15%), while a score of 6 indicates the most severe regional ventilation defects (three quadrants with <10% ventilation and one quadrant receiving the vast majority of ventilation).

The global inhomogeneity index (GI) was used to assess the degree of inhomogeneity in global ventilation distribution. It was defined as the sum of the absolute differences between the tidal impedance variation of each lung pixel and the median tidal impedance variation of all lung pixels, divided by the sum of tidal impedance variation in all lung pixels. GI ranges from 0 (perfect homogeneity) upward, with no mathematically fixed upper limit; values >1.0 have been reported in the literature under conditions of severe ventilation inhomogeneity (e.g., one-lung ventilation) (Zhao et al., 2009).


$$GI=\frac{\sum_{i\in \Omega }|\Delta Z_{i}-median(\Delta Z_{\Omega })|}{\sum_{i\in \Omega }\Delta Z_{i}}$$

where Ω represents all pixels within the lung region, and $\Delta Z_{i}\;$represents the tidal impedance variation of pixel i.

The center of ventilation (CoV) was calculated as the percentage of ventilation distribution along the ventral–to–dorsal axis, ranging from 0% (ventral) to 100% (dorsal), where 50% indicates perfect ventral–dorsal symmetry (Frerichs et al., 2008; Frerichs et al., 2020; Sun et al., 2026). In this study, CoV was calculated only along the ventral-to-dorsal axis. CoV along the right-to-left axis was not calculated because the primary objective was to evaluate gravity-dependent ventilation redistribution and regional derecruitment after BAL in supine mechanically ventilated patients, rather than lateral ventilation asymmetry. A higher CoV value indicated a shift of ventilation toward the dorsal lung regions, whereas a lower CoV value indicated a shift toward the ventral lung regions.


$$CoV(\%)=\frac{\sum_{i\in \Omega }y_{i}\Delta Z_{i}}{H\sum_{i\in \Omega }\Delta Z_{i}}\times 100\%$$

where $\;y_{i\;}$ represents the coordinate of pixel *i* along the ventral-to-dorsal axis, and *H* represents the total height of the EIT lung region along this axis.

Regional ventilation delay (RVD) was calculated as the time required for each pixel to reach 40% of its maximum inspiratory impedance change (Wrigge et al., 2008; Muders et al., 2019). This value was normalized to the global inspiratory time, and the standard deviation was then calculated. As a standard deviation, RVD is a non-negative value (≥0) with no fixed upper limit; in clinical practice, it typically remains below 50%. A higher RVD indicated greater asynchrony in regional ventilation, suggesting more marked ventilation delay and abnormal ventilation distribution.


$$RVD=SD(\frac{t_{i,40\%}-t_{global,40\%}}{T_{insp}})\times 100\%$$

where $t_{i,40\%}$ represents the time required for pixel *i* to reach 40% of its maximum inspiratory impedance change, $t_{global,40\%}$ represents the time required for the global lung impedance curve to reach 40% of its maximum inspiratory impedance change, and $T_{insp}$ represents the inspiratory time.

### 2.5 Study outcomes

The primary outcome was the degree of immediate lung derecruitment after BAL. It was expressed as the absolute and relative change in EELI at T3 compared with T1:


$$\Delta EELI_{T3-T1}(\%)=\frac{EELI_{T3}-EELI_{T1}}{\left|EELI_{T1}\right|}\times 100\%$$

These parameters were used to assess the change in lung volume after BAL compared with baseline. A greater decrease in EELI indicated more severe BAL-associated lung volume loss and lung derecruitment. For easier clinical interpretation, residual EELI loss was further defined as follows:


$$ResidualEELIloss(\%)=\frac{EELI_{T1}-EELI_{T3}}{\left|EELI_{T1}\right|}\times 100\%$$

When EELI at T3 was lower than that at T1, residual EELI loss (%) was positive. This indicated persistent lung volume loss after completion of BAL and withdrawal of the bronchoscope. A higher value suggested more severe residual BAL-associated lung derecruitment.

The secondary outcomes included the following.

First, dynamic changes in periprocedural EIT parameters were assessed. EELI, DS, GI, CoV, and RVD were compared among T1, T2, and T3. These comparisons were used to evaluate immediate changes in lung volume, ventilation defects, and regional ventilation distribution during BAL.

Second, dynamic changes in periprocedural respiratory mechanics were assessed. Tidal volume, peak airway pressure, plateau pressure, driving pressure, dynamic compliance, SpO_2_, and end-tidal carbon dioxide were compared among T1, T2, and T3. These comparisons were used to evaluate the effects of BAL on global respiratory mechanics and gas exchange.

Third, factors associated with residual lung derecruitment after BAL were analyzed. Patient baseline characteristics, preoperative CT findings, baseline EIT parameters, baseline respiratory mechanics, and BAL-related procedural variables were included to identify clinical, radiological, and physiological factors associated with residual EELI loss (%).

### 2.6 Statistical analysis

Data were analyzed using SPSS version 29.0. Continuous variables were first tested for normality. Normally distributed variables are presented as mean ± standard deviation, and non-normally distributed variables are presented as median and interquartile range. Categorical variables are presented as number and percentage. Descriptive statistics summarized baseline characteristics, lung diseases, CT findings, EIT parameters, respiratory mechanics, and BAL procedural data.

For EIT parameters and respiratory mechanics measured at T1, T2, and T3, repeated-measures analysis of variance was used to assess the time effect. When the assumption of sphericity was not met, the Greenhouse–Geisser correction was applied. For repeated-measures data that did not meet the assumptions for parametric testing, the Friedman test was used. If the overall comparison was statistically significant, pairwise comparisons were further performed, including T2 versus T1, T3 versus T1, and T3 versus T2. Bonferroni correction was used for multiple comparisons. These analyses were used to evaluate dynamic changes in lung volume, regional ventilation distribution, and respiratory mechanics during the periprocedural period of BAL.

Factors associated with residual EELI loss (%) after BAL were analyzed using linear regression. Univariable analysis was first performed for age, sex, BMI, underlying lung diseases, CT findings, baseline EIT parameters, baseline respiratory mechanics, total BAL instilled volume, number of BAL cycles, and bronchoscope insertion duration. Variables were selected for multivariable analysis based on univariable results, clinical relevance, and collinearity; representative variables were retained when variables had similar meaning or strong correlations to avoid overfitting and multicollinearity.

All tests were two-sided, with P < 0.05 considered statistically significant.

## 3 Results

**Figure 2:**
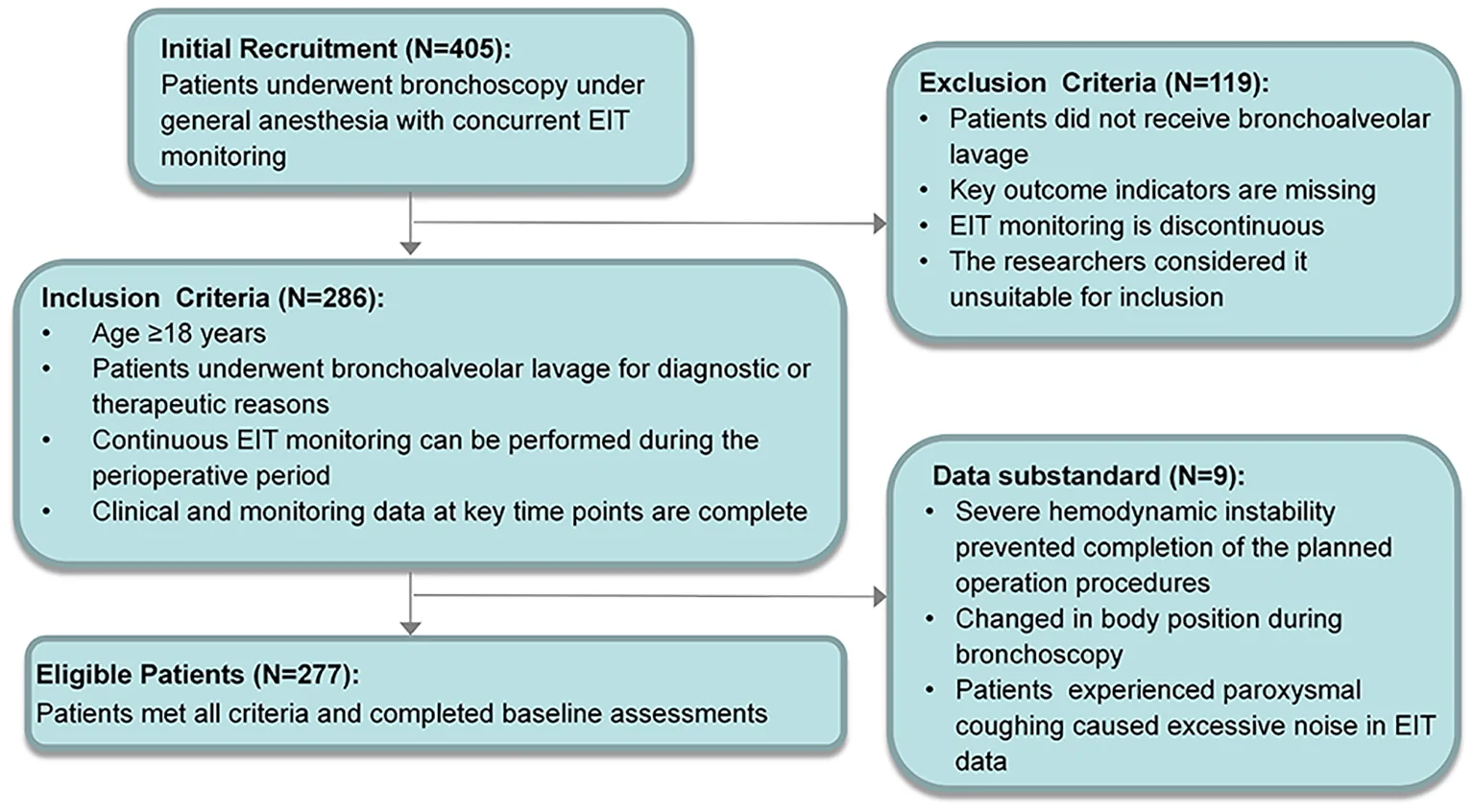
Study flowchart. The diagram illustrates the patient selection process, including screening and exclusion criteria, 277 patients were included in the final analysis.

The patient selection process and baseline characteristics are shown in **Figure 2** and **Table 1**. During the study period, 405 patients underwent bronchoscopy under general anesthesia with EIT monitoring. Of these, 286 patients met the inclusion criteria. After exclusion of patients with invalid EIT data, 277 patients were included in the final analysis. Overall, the cohort mainly consisted of middle-aged and older male patients. Lung cancer and abnormal preoperative CT findings were common. All patients underwent BAL and EIT monitoring under general anesthesia and mechanical ventilation. Periprocedural SpO_2_ was maintained at 100%.

**Table 1:** Baseline and procedural characteristics of the study cohort.

Variable	Total cohort, n=277
Demographic characteristics	
Age, years	58.55 (53.00, 68.00)
Male sex, n (%)	176 (63.54)
BMI, kg/m²	22.98 (20.31, 25.71)
Underlying pulmonary conditions and CT findings	
COPD/asthma, n (%)	34 (12.30)
Lung cancer, n (%)	136 (49.10)
Interstitial lung disease/pulmonary fibrosis, n (%)	19 (6.86)
Pre-BAL CT atelectasis, n (%)	121 (43.70)
Pre-BAL CT consolidation/infiltration, n (%)	112 (40.43)
Pre-BAL CT emphysema/bullae/cavity, n (%)	35 (12.64)
Pre-BAL arterial blood gas analysis	
Arterial pH	7.42 (7.39, 7.44)
PaO_2_, mmHg	79.00 (71.00, 85.00)
PaCO_2_, mmHg	39.19 ± 4.49
Baseline ventilatory status before BAL	
Tidal volume, mL	448.00 (399.00, 473.00)
Plateau pressure, cmH_2_O	12.00 (10.00, 14.00)
Peak airway pressure, cmH_2_O	15.00 (12.00, 17.00)
Driving pressure, cmH_2_O	10.00 (8.00, 12.00)
Dynamic compliance, mL/cmH_2_O	42.55 (33.92, 52.22)
Minute ventilation, L/min	6.00 (5.10, 6.70)
End-tidal CO_2_, mmHg	33.00 (30.00, 35.00)
Baseline EIT-derived variables before BAL	
DS, (0-6)	2.0 (1.5, 4.0)
GI, (>0)	0.70 (0.54, 0.99)
CoV, ventral %, (0-100%)	44.7 (39.2, 50.2)
RVD, (≥0)	16.7 (12.2, 21.1)
Bronchoscopy procedural characteristics	
Total instilled BAL volume, mL	75.00 (30.00, 75.00)
Number of BAL cycles, counts	5.00 (2.00, 5.00)
Bronchoscope insertion duration, min	23.00 (16.00, 33.00)

Continuous variables are shown as mean ± standard deviation or median (interquartile range). Categorical data are numbers (%). BMI, body mass index; COPD, chronic obstructive pulmonary disease; BAL, bronchoalveolar lavage; PaO_2_, partial pressure of oxygen in arterial blood; PaCO_2_, partial pressure of carbon dioxide in arterial blood; DS, defect score; GI, global inhomogeneity index; CoV, center of ventilation; RVD, regional ventilation delay.

The actual intervals between the EIT measurements and the BAL procedure showed slight variation from the prespecified target intervals. The median interval between T1 and the initiation of BAL was 10.2 min (IQR, 8.5–12.5 min). T2 was recorded at a median of 1.3 min after BAL completion (IQR, 0.5–2.1 min), while the bronchoscope remained in place. T3 was recorded at a median of 20.0 min after BAL completion (IQR, 14.0–28.0 min), after bronchoscope removal and during continued mechanical ventilation. These variations were related to differences in cardiorespiratory stabilization, procedural duration, bronchoscope removal, and EIT signal acquisition under clinical conditions.

Periprocedural changes in EIT parameters and respiratory mechanics are shown in **Table 2**. Compared with T1, EELI decreased significantly after BAL. ΔEELI decreased from 0.00% at baseline to −0.66% (−1.45%, −0.17%) at T2 (P<0.001). At T3, after continued mechanical ventilation, ΔEELI further decreased to −1.26% (−2.91%, −0.54%), with significant differences compared with both T1 and T2 (both P<0.001). Regional ventilation distribution also became more heterogeneous after BAL. DS increased from 2.0 (1.5, 4.0) at T1 to 3.0 (2.0, 4.0) at both T2 and T3. GI increased from 0.70 (0.54, 0.99) at T1 to 0.83 (0.61, 1.16) at T2 and 0.77 (0.58, 1.12) at T3 (all P<0.001). RVD did not change significantly at T2, but it increased significantly at T3 compared with both T1 and T2, suggesting impaired synchrony of regional ventilation.

**Table 2:** Periprocedural changes in EIT-derived variables and respiratory mechanics during BAL.

Variable	T1	T2	T3	Overall P value	T2 vs T1	T3 vs T1	T3 vs T2
EIT-derived variables							
ΔEELI from T1, %	0.00 (0.00, 0.00)	−0.66 (−1.45, −0.17)	−1.26 (−2.91, −0.54)	<0.001	<0.001	<0.001	<0.001
DS (0-6)	2.0 (1.5, 4.0)	3.0 (2.0, 4.0)	3.0 (2.0, 4.0)	<0.001	<0.001	<0.001	0.073
GI (>0)	0.70 (0.54, 0.99)	0.83 (0.61, 1.16)	0.77 (0.58, 1.12)	<0.001	<0.001	<0.001	0.319
CoV, ventral % (0-100%)	44.7 (39.2, 50.2)	43.6 (38.3, 50.8)	43.4 (37.5, 50.6)	<0.001	<0.001	<0.001	1.000
RVD (≥0)	16.7 (12.2, 21.1)	16.7 (12.9, 21.5)	18.6 (13.6, 22.7)	<0.001	0.585	<0.001	0.003
Respiratory mechanics and gas exchange							
Peak airway pressure, cmH_2_O	15.0 (12.0, 17.0)	19.0 (15.0, 23.0)	19.0 (15.0, 23.0)	<0.001	<0.001	<0.001	0.808
Driving pressure, cmH_2_O	10.0 (8.0, 12.0)	11.0 (9.0, 15.0)	12.0 (10.0, 15.0)	<0.001	<0.001	<0.001	<0.001
Dynamic compliance, mL/cmH_2_O	42.5 (33.9, 52.2)	26.2 (17.6, 34.9)	32.7 (25.7, 43.3)	<0.001	<0.001	<0.001	<0.001
End-tidal CO_2_, mmHg	33.0 (30.0, 35.0)	29.0 (26.0, 32.0)	33.0 (30.0, 37.0)	<0.001	<0.001	0.118	<0.001

Continuous variables are shown as median (interquartile range). A P-value<0.05 was considered statistically significant. T1, measured after anesthesia stabilization, before BAL (median interval to BAL start: 10.2 min); T2, immediately post-BAL (median BAL duration: 1.3 min); T3, immediately after scope removal (median interval from BAL end: 20.0 min). EIT, electrical impedance tomography; BAL, bronchoalveolar lavage; ΔEELI, change in end-expiratory lung impedance; DS, defect score; GI, global inhomogeneity index; CoV, center of ventilation; RVD, regional ventilation delay.

**Figure 3:**
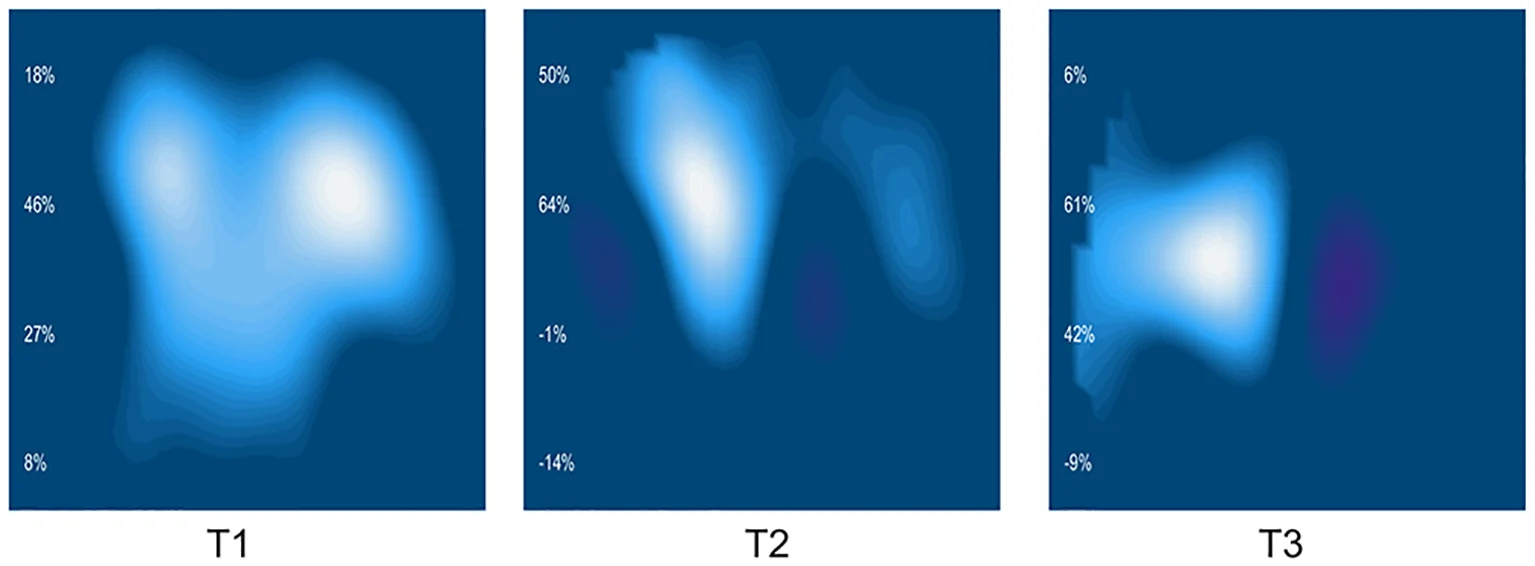
Representative EIT ventilation maps from one patient. The images show the distribution of tidal ventilation at the three times (T1–T3). Colors represent the magnitude of regional tidal impedance change, with bright areas indicating high ventilation and dark blue areas indicating low or no ventilation. The progressive increase in blue (low ventilation) areas from T2 to T3 illustrates the development of ventilation decreased or disappeared in some lung regions after bronchoalveolar lavage.

In terms of respiratory mechanics, peak airway pressure and driving pressure increased after BAL, while dynamic compliance decreased. Peak airway pressure increased from 15.0 (12.0, 17.0) cmH_2_O at T1 to 19.0 (15.0, 23.0) cmH_2_O at both T2 and T3. Driving pressure increased from 10.0 (8.0, 12.0) cmH_2_O at T1 to 11.0 (9.0, 15.0) cmH_2_O at T2 and 12.0 (10.0, 15.0) cmH_2_O at T3 (all P<0.001). Dynamic compliance decreased from 42.5 (33.9, 52.2) mL/cmH_2_O at T1 to 26.2 (17.6, 34.9) mL/cmH_2_O at T2. Although it partially recovered to 32.7 (25.7, 43.3) mL/cmH_2_O at T3, it remained significantly lower than the baseline value at T1 (P<0.001). **Figure 3** visually shows that ventilation decreased or disappeared in some lung regions after BAL, with only partial recovery at T3. These findings were consistent with the decrease in EELI, the increases in DS and GI, and the reduction in dynamic compliance shown in **Table 2**.

**Figure 4:**
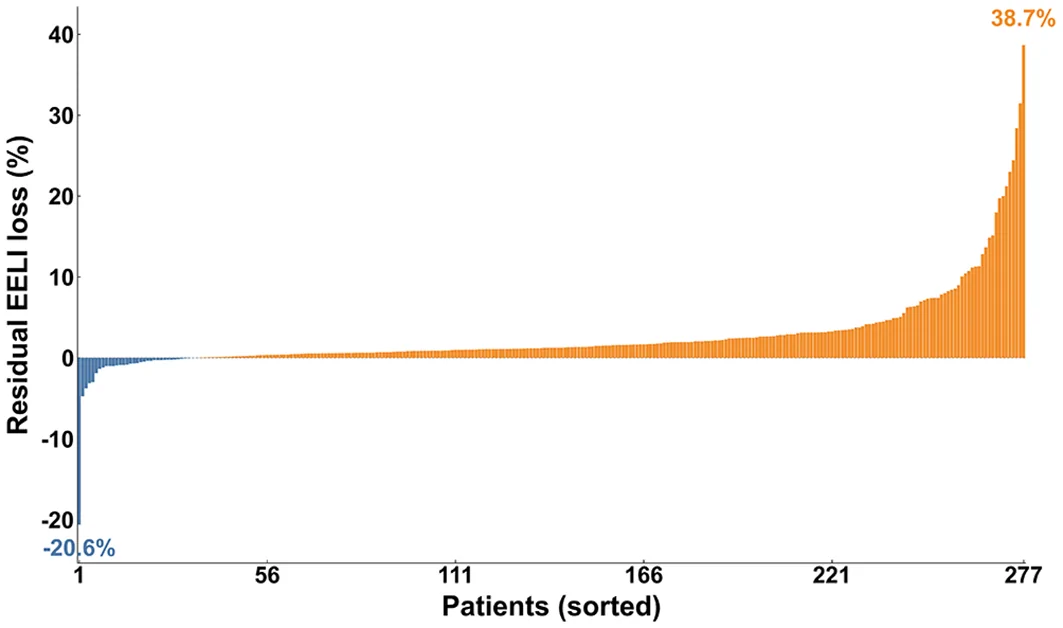
The individual distribution of residual EELI loss after bronchoalveolar lavage. EELI, end-expiratory lung impedance.

**Figure 4** shows the individual distribution of residual EELI loss after BAL. Most patients had positive residual EELI loss, indicating persistent EELI reduction compared with baseline. A small proportion of patients showed negative values, suggesting an increase in EELI after BAL. The waterfall plot also showed marked inter-individual variation. While most patients had only mild residual EELI loss, a subset showed a large decrease in EELI.

Factors associated with residual EELI loss are shown in **Table 3**. In the univariable analysis, BMI, chronic obstructive pulmonary disease (COPD)/asthma, preoperative atelectasis on CT, and baseline DS were associated with residual EELI loss. In contrast, smoking history, lung cancer, interstitial lung disease/pulmonary fibrosis, preoperative consolidation or infiltration on CT, emphysema/bullae/cavity, baseline dynamic compliance, end-tidal carbon dioxide, and procedural variables were not significantly associated with residual EELI loss. Multivariable analysis further showed that preoperative atelectasis on CT and baseline DS were independently associated with residual EELI loss. Patients with preoperative atelectasis had a 1.71-percentage-point greater residual EELI loss than those without atelectasis (95% CI: 0.41–3.00, P = 0.010). Each 1-point increase in baseline DS was associated with a 0.51-percentage-point increase in residual EELI loss (95% CI: 0.07–0.95, P = 0.025). In addition, COPD/asthma was associated with lower residual EELI loss (β=−2.28, 95% CI: −4.11 to −0.45, P = 0.015).

**Table 3:** Univariable and multivariable linear regression analyses of factors associated with residual EELI loss after BAL.

Variable	Univariable β coefficient (95% CI)	P value	Multivariable adjusted β coefficient (95% CI)	P value
Age, years	0.02 (−0.02, 0.07)	0.295	—	—
Male sex	−0.37 (−1.66, 0.92)	0.575	—	—
BMI, kg/m²	0.20 (0.03, 0.37)	**0.023**	0.12 (−0.05, 0.29)	0.157
Smoke	0.08 (−1.20, 1.36)	0.909	—	—
COPD/asthma	−2.21 (−4.09, −0.33)	**0.022**	−2.28 (−4.11, −0.45)	**0.015**
Lung cancer	0.73 (−0.52, 1.97)	0.252	—	—
Interstitial lung disease/pulmonary fibrosis	1.95 (−0.50, 4.41)	0.120	—	—
Pre-BAL CT atelectasis	2.23 (1.00, 3.46)	**<0.001**	1.71 (0.41, 3.00)	**0.010**
Pre-BAL CT consolidation/infiltration	0.02 (−1.25, 1.28)	0.981	—	—
Pre-BAL CT emphysema/bullae/cavity	−0.02 (−1.90, 1.85)	0.982	—	—
Baseline defect score	0.67 (0.25, 1.09)	**0.002**	0.51 (0.07, 0.95)	**0.025**
Baseline dynamic compliance, mL/cmH_2_O	0.00 (−0.03, 0.04)	0.857	—	—
Baseline End-tidal CO_2_, mmHg	−0.08 (−0.23, 0.07)	0.297	—	—
Total instilled BAL volume, mL	0.00 (−0.02, 0.03)	0.883	—	—
Number of BAL cycles, counts	0.03 (−0.35, 0.41)	0.883	—	—
Bronchoscope insertion duration, min	0.02 (−0.03, 0.07)	0.409	—	—

A P-value<0.05 was considered statistically significant. EELI, end-expiratory lung impedance; BAL, bronchoalveolar lavage; BMI, body mass index; COPD, chronic obstructive pulmonary disease.

Bold values indicate statistical significance (P < 0.05) in the multivariable linear regression analysis. Variables were selected for the multivariable model based on univariable significance (P < 0.10), clinical relevance, and collinearity assessment.

## 4 Discussion

In this study, EIT monitored patients during BAL under general anesthesia and mechanical ventilation. We found that BAL was associated with clear lung derecruitment and worsening regional ventilation distribution, as reflected by decreased EELI and increased DS and GI. These changes were accompanied by reduced dynamic compliance and increased driving pressure. Notably, despite both EIT and respiratory mechanics indicating impaired ventilation, SpO_2_ remained high and stable at 100% throughout the periprocedural period. This suggests that, under high inspired oxygen and mechanical ventilation, conventional oxygenation parameters may not fully reflect BAL-related regional lung volume loss and ventilation inhomogeneity, and EIT may provide more direct information on regional ventilation.

Several mechanisms may explain these findings. Instilled lavage fluid may transiently occupy alveolar and small airway spaces, and may dilute or remove local surfactant, promoting alveolar closure (Soliman et al., 2021). Meanwhile, bronchoscope insertion may increase airway resistance, reduce effective airway lumen, and alter gas distribution during mechanical ventilation. These factors may reduce ventilation in some lung units or cause airway and alveolar closure (Pritchett et al., 2021), reflected by decreased EELI and increased ventilation defects. Once derecruitment occurs, fewer lung units participate in tidal ventilation, requiring higher pressure to deliver the same tidal volume (Beitler, 2020), which explains the increased driving pressure and decreased dynamic compliance. The consistent direction of EIT and respiratory mechanics changes supports the presence of regional derecruitment and impaired mechanics after BAL. The lack of a clear decrease in SpO_2_ may be due to high oxygen reserve, high FiO_2_ during anesthesia, and compensatory ventilation in non-lavaged or better-ventilated regions, suggesting that stable SpO_2_ does not exclude occult regional ventilation impairment.

The waterfall plot confirms the heterogeneity of BAL-associated lung derecruitment. Although mean EELI decreased after BAL, individual responses varied widely, ranging from little change or even an increase to marked residual loss. This indicates that BAL effects are not uniform across patients, and may depend on baseline lung condition, regional ventilation reserve, airway closure tendency, and the recruitability of unstable lung units during mechanical ventilation. Thus, group-level EELI changes should be interpreted alongside individual response patterns, reinforcing the utility of EIT as a bedside tool to detect patient-specific ventilation changes during bronchoscopy.

The analysis showed that preoperative atelectasis on CT and a higher baseline DS were independently associated with residual EELI loss after BAL, suggesting that BAL-related derecruitment is more likely in patients with pre-existing regional ventilation impairment (Borgmann et al., 2025). Patients with atelectasis may have poor alveolar stability and more low-ventilation regions, making them more sensitive to lavage fluid, transient airway obstruction, and ventilation changes (Bachmann et al., 2025). A higher baseline DS indicates more severe pre-existing ventilation defects, with fewer recruitable units available and limited recovery after lavage, thus predisposing to residual EELI loss. In contrast, total BAL volume, number of cycles, and bronchoscope insertion duration were not significantly associated with residual EELI loss, indicating that, within the procedural range of this study, baseline lung condition may be more important than procedural dose or duration in determining residual derecruitment after BAL.

Interestingly, COPD/asthma showed a negative association with residual EELI loss, though this finding requires cautious interpretation. These obstructive airway diseases are characterized by airflow limitation, gas trapping, and dynamic hyperinflation (Huang et al., 2016). Under mechanical ventilation, such patients may have higher baseline end-expiratory lung volume, so the relative percentage reduction in EELI could appear smaller. Moreover, the primary pathophysiology in COPD/asthma involves increased airway resistance, expiratory flow limitation, and regional ventilation delay, rather than alveolar collapse per se (Papapostolou et al., 2025; Singh et al., 2025). Thus, BAL-induced changes may not be adequately captured by EELI loss alone. The potential confounding effects of bronchodilators, inhaled therapy, or more conservative ventilatory settings cannot be excluded. Since we did not stratify COPD/asthma by severity, reversibility, medication use, or degree of hyperinflation, this association remains exploratory and awaits confirmation in future studies.

This study has several limitations. First, it was a single-center retrospective design, which may introduce selection bias and unmeasured confounding, and precludes causal inference. Second, BAL procedures, lavage sites, suction intensity, ventilation settings, and anesthesia management followed real-world clinical practice and were not fully standardized, potentially affecting EIT and respiratory measurements. Third, only short-term periprocedural changes were evaluated; longer-term follow-up was unavailable, so we could not determine how long residual EELI loss persisted or whether it was associated with postoperative pulmonary complications. Finally, information on disease subtypes and severity was limited (e.g., COPD/asthma was not stratified). These findings should be confirmed in prospective multicenter studies.

## 5 Conclusion

Under general anesthesia and mechanical ventilation, BAL was associated with EIT-derived decreases in EELI, increased ventilation defects, and greater ventilation inhomogeneity. These changes were accompanied by increased driving pressure and reduced dynamic compliance, suggesting BAL-associated lung derecruitment. Although SpO_2_ remained stable and close to 100% during the periprocedural period, these EIT and respiratory mechanics changes were still observed after BAL. This indicates that conventional oxygenation parameters may underestimate regional ventilation impairment. EIT may help assess BAL-associated lung derecruitment and identify related high-risk factors, providing useful information for individualized respiratory management during bronchoscopy.

## Data Availability

The raw data supporting the conclusions of this article will be made available by the authors, without undue reservation.
