# Supplementary material for: Electrical impedance tomography assessment of bronchoalveolar lavage-associated lung derecruitment and its related factors: a single-center retrospective study
Source: Front Physiol. 2026 Sep 4;17:1900825. doi: 10.3389/fphys.2026.1900825 (PMC13585634; doi:10.3389/fphys.2026.1900825)
Supplement: Supplementary file 1 [file Table1.docx]

**Table S1. Comparison of EIT parameters at T1, T2, and T3 between patients with and without COPD/asthma**

| **Variable** | **Group** | **T1** | **T2** | **T3** | **Overall P value** |
| --- | --- | --- | --- | --- | --- |
| **EIT-derived variables** |  |  |  |  |  |
| ΔEELI from T1, % | non-COPD/Asthma  (n = 243) | 0.00  (0.00, 0.00) | −0.73  (−1.50, −0.26) | −1.42  (−3.14, −0.65) | **0.004** |
|  | COPD/Asthma  (n = 34) | 0.00  (0.00, 0.00) | −0.16  (−0.58, 0.25) | −0.42  (−1.10, 0.59) |  |
| DS  (0-6) | non-COPD/Asthma  (n = 243) | 2.0  (1.0, 4.0) | 3.0  (2.0, 4.0) | 3.0  (2.0, 4.0) | 0.277 |
|  | COPD/Asthma  (n = 34) | 2.00  (2.00, 4.00) | 3.5  (2.0, 4.0) | 4.00  (2.00, 4.00) |  |
| GI  (>0) | non-COPD/Asthma  (n = 243) | 0.69  (0.54, 0.99) | 0.83  (0.59, 1.15) | 0.76  (0.58, 1.13) | 0.340 |
|  | COPD/Asthma  (n = 34) | 0.74  (0.53, 0.93) | 0.88  (0.67, 1.23) | 0.79  (0.58, 1.02) |  |
| CoV, ventral %  (0-100%) | non-COPD/Asthma  (n = 243) | 44.9  (39.4, 50.2) | 43.7  (38.0, 51.2) | 43.7  (37.8, 51.1) | 0.773 |
|  | COPD/Asthma  (n = 34) | 43.8  (38.6, 48.6) | 41.9  (38.7, 45.6) | 40.2  (37.0, 46.1) |  |
| RVD  (≥0) | non-COPD/Asthma  (n = 243) | 17.1  (12.1, 21.6) | 16.7  (12.9, 21.5) | 18.7  (13.3, 22.7) | 0.242 |
|  | COPD/Asthma  (n = 34) | 16.6  (13.3, 20.7) | 17.4  (13.5, 21.1) | 17.4  (14.9, 21.8) |  |

Data are presented as median (interquartile range). P values were calculated using the generalized linear models for between-group comparisons at each time point. T1, measured after anesthesia stabilization, before BAL (median interval to BAL start: 10.2 min); T2, immediately post‑BAL (median BAL duration: 1.3 min); T3, immediately after scope removal (median interval from BAL end: 20.0 min). Abbreviations: EIT: electrical impedance tomography; BAL: bronchoalveolar lavage; EELI: end-expiratory lung impedance; DS: defect score; GI: global inhomogeneity index; CoV: centre of ventilation; RVD: regional ventilation delay.
